# A global resource to translational medicine: the International Park of Translational Medicine and BioMedicine (IPTBM)

**DOI:** 10.1186/1479-5876-11-8

**Published:** 2013-01-08

**Authors:** Xiaodan Wu, Francesco M Marincola, Michael N Liebman, Xiangdong Wang

**Affiliations:** 1 Department of Respiratory Medicine, Fudan University, Shanghai, China; 2 Section of Infectious Disease and Immunogenetics, Department of Transfusion Medicine, Clinical Center, National Institutes of Heath, Bethesda, MD, USA; 3 Biomedical research Center, Zhongshan Hospital, Shanghai Medical College, Fudan University, Shanghai, China; 4 IPQ Analytics, LLC, Bethesda, MD, USA; 5 Strategic Medicine, Inc, Bethesda, MD, USA

**Keywords:** Translational medicine, International, Projects, Development, Management, Strategy

## Abstract

Translational science consists of research and development that integrates multiple resources to expedite the successful treatment of disease. The International Park of Translational BioMedicine (IPTBM) is currently being developed within the interface between Zhejiang Province and Shanghai Municipality. IPTBM has been designed to pioneer comprehensive biomedical research that spans the continuum from the education of young scientists to providing the infrastructure necessary for clinical testing and direct observation to better understand human biology while promoting viable commercial results within a vibrant biotechnology community. IPTBM’s goal is to attract global partners organized around five fundamental pillars: 1) Institutional Development, 2) Project Implementation, 3) Development and Production, 4) Investment and 5) Regulatory Clusters to address the needs of an international platform of scientists, institutes, universities, commercial enterprises, investors, politicians, and other stakeholders. The IPTBM differs from existing models including CTSA’s (US, NIH) technology because of its comprehensive approach to merge education, research, innovation, and development to translate clinical and public health needs into target-oriented and cost-efficient projects.

## 

A big challenge exists in trying to translate scientific ideas into actual experiments, the results of basic research into clinical application, products into commercial value, and research results into policies and regulations. It is also challenging to expand the development of translational science to learn the limitations and potentials of current treatment approaches from direct human observation. Translational research has been defined Bench to Bedside but where the need for the process to extend also from Bedside to Bench, creating a two-way path, was emphasized a decade ago by Marincola [1]. As a result, the process of translational science needs to include not only the insular participation of scientists, clinicians, and universities, but also commercial organizations, ethicists, regulatory agencies, and other entities to collaborate towards guarantee the most effective, useful and safe approach to produce benefit to mankind. The International Park of Translational BioMedicine (IPTBM) was planned and it is scheduled to be launched in January of 2013 in Southern China within the interface between the Zhejiang Province and Shanghai Municipality; IPTBM is one of several research and development parks within the new area of innovation and development known as Sino Science & Technology Park of Zhangjiang-Hangzhou Bay. It consists of 65 square kilometers and it is sponsored by both local and central government resources. The Park aims to gather global scientific resources that will include individual scientists, academic institutions, entrepreneurs, investors, and regulatory experts in translational biomedicine, in order to create an international platform that spans the continuum comprising education, innovation, development, marketing, regulation and, investment. The Park will accomplish this goal by establishing a new, environmentally sound, high-technology, collaboration-based community.

The IPTBM has been designed to learn from the collected experiences of two major efforts, the creation of Clinical Translational Science Awards (Centers), CTSA, by the National Institutes of Health in the United States, and in the global development of technology parks. As part of the NIH Roadmap for Medical Research [2], institutions were encouraged to reengineer resources and programs of clinical and translational research. The Clinical and Translational Science Awards (CTSA) program was created and is currently sponsored by the National Center for Advancing Translational Sciences at the NIH. The CTSA program attempted to reengineer the flow of resources to strengthen the full spectrum of translational research and improve the quality and efficiency of the process. IPTBM will also identify and reengineer current resources associated with translational research to increase and optimize the value of translational experts and centers [3]. Beyond the scope of the CTSA program, however, the IPTBM plans to attract and gather global powers and resources organized around five fundamental pillars defined as 1) Institutional Development, 2) Project Implementation, 3) Development and Production, 4) Investment and 5) Regulatory Clusters. Institutional Development Clusters will group institu-tions of translational biomedicine owned, sponsored, or in partnership with internationally recognized universities, schools, or enterprises. The Project Clusters will foster individual investigator-generated projects with the potential for directed application and commercialization. Development & Production Clusters, the downstream facet of translational research, will include companies supported by the common aggregation of infrastructure, facilities, and production capacities. Clusters for investments and for regulatory groups will provide financial support from private and public sectors, administrative assistance from local and national management, and facilitate commercial development and marketing.

IPTBM also aspires to develop an advanced plat-form for graduate education and become a professional incubator generating future-leading scientists in translational biomedicine. Zerhouni emphasized the importance and difficulties of recruiting, mentoring, and retaining an international assembly of qualified clinical and translational scientists who can conduct truly innovative patient-oriented research and development, work under the pressures of delivery demands, and perform outstanding research efficiently leading to an optimal end result [4]. Unlike other existing technology parks, IPTBM will provide educational programs in clinical and translational research offering graduate degrees under the supervision of internationally recognized faculty and mentors. The Institutional Clusters are expected to identify recognized and qualified global level mentors to assure the quality of the educational research program [5]. The Institutes in IPTBM will assume full responsibility for the training of clinical and translational scientists and the assessment and development of relevant disciplines and sub-disciplines.

IPTBM intends to provide the port where translational research projects can softly land and be transferred through the critical phases of development and regulatory application, especially in China. IPTBM can foster and accelerate projects exhibiting the potential for commercial application, generated by the Institute of Translational Biomedicine and individual scientists, early phase enterprises, or licensing sectors, through their maturation or late phase development and even into production or commercialization. Different from CTSAs and business incubators like science parks, the IPTBM with its Institutional Clusters will provide the full spectrum of requirements for effective biomedical translation. IPTBM will assist scientists, institutes, and companies in the development of the reputation and market recognition of the projects and/or products, evaluation and recruitment of development budgets and investments, or minimization and avoidance of possible risks and damages during development and marketing. Criteria to measure the success of IPTBM will include business objectives, rates of successful conversion from research projects to commercial application, expansion of a high quality scientific community, and the development of education and innovation projects.

The IPTBM will play a comprehensive role in driving biomedical investigation. CTSAs generally support infrastructure and resources for clinical research, provide a national consortium of medical research institutions that can work together to improve clinical and translational research and enhance the efficiency and quality. Innovation science and technology parks or incubators mainly provide infrastructure and administrative assistance to foster developing scientific ideas into projects with commercial potential and help the initial formation of commercial companies. The IPTBM will establish a centralized entity in which the clusters that combine the components relevant to translational research can converge to integrate education, innovative research and development, project-oriented activities, and application-dominated strategies that will also require business development, external investment, and regulation. It is different from the concept of clinical and translational medicine as integrative and practical science which integrates clinical research with modern methodologies in systems and computational biology, and high-throughput analyses [6,7]. For example, clinical and translational medicine can foster the implementation of human tissue banking, and the development of bio-banks linked to high quality clinical data bases, for identification of clear phenotypes relevant to stratification of patients receiving standard or experimental therapies. However, the Park will act as the key player to trigger innovation and project development, to help and develop subsidiaries from existing organizations as peninsulas rather than as islands and as extensions of the company’s strategic domain rather than as isolated outposts [8]. The Park will encourage both formal and informal channels of communication between corporate activities of parent companies and their subsidiaries and to give subsidiaries more authority to carry their ideas through to completion. The Park will seed economic support to scientists, institutes, early stage projects, and corporate subsidiaries, be attractive to investors and financial supporters not only for research and development of projects, but also for education and investigation of clinical needs, development of translational principles, and establishment of public health policies and regulations. However, there are many more challenges that the IPTBM has to face and solve, such as a fragmented infrastructure, lack of adequately trained investigators, bureaucratic and regulatory burdens, or lack of support by the public [9].

In conclusion, research and development in translational science will integrate scientists, clinicians, universities, companies, ethical and advisory committees, governmental regulatory agencies, and, indirectly, governments. The International Park of Translational BioMedicine is designed to lead this integrative approach to create a vibrant international community of experts among the varied critical field relevant to effective biomedical translation.
